# Hydrogen Sulfide in Plants: Crosstalk with Other Signal Molecules in Response to Abiotic Stresses

**DOI:** 10.3390/ijms222112068

**Published:** 2021-11-08

**Authors:** Chunlei Wang, Yuzheng Deng, Zesheng Liu, Weibiao Liao

**Affiliations:** College of Horticulture, Gansu Agricultural University, Lanzhou 730070, China; wangchunlei@gsau.edu.cn (C.W.); dengyz0830@163.com (Y.D.); lzs0724@163.com (Z.L.)

**Keywords:** hydrogen sulfide, nitric oxide, abscisic acid, Ca^2+^, hydrogen peroxide, abiotic stresses, signal transmitters, stomatal movement

## Abstract

Hydrogen sulfide (H_2_S) has recently been considered as a crucial gaseous transmitter occupying extensive roles in physiological and biochemical processes throughout the life of plant species. Furthermore, plenty of achievements have been announced regarding H_2_S working in combination with other signal molecules to mitigate environmental damage, such as nitric oxide (NO), abscisic acid (ABA), calcium ion (Ca^2+^), hydrogen peroxide (H_2_O_2_), salicylic acid (SA), ethylene (ETH), jasmonic acid (JA), proline (Pro), and melatonin (MT). This review summarizes the current knowledge within the mechanism of H_2_S and the above signal compounds in response to abiotic stresses in plants, including maintaining cellular redox homeostasis, exchanging metal ion transport, regulating stomatal aperture, and altering gene expression and enzyme activities. The potential relationship between H_2_S and other signal transmitters is also proposed and discussed.

## 1. Introduction

Several abiotic stresses such as salt, drought, flooding, heat, cold, and freezing easily result in the loss of crop production and a drop in economy in the world. Furthermore, with ongoing industrialization and pesticides application, plants are more likely subjected to some abiotic stresses including salinity and heavy metal (aluminum (Al); cadmium (Cd); chromium (Cr); lead (Pb); cobalt (Co); arsenic (As); nickel (Ni)) stresses [1,2]. In order to survive, plants must make a series of adjustments in morphology and physiological and biochemical metabolism when they are subjected to abiotic stresses. There are many kinds of mechanisms for plants to respond to abiotic stresses, including plant hormones, osmotic regulators, active oxygen scavenging systems, genes, and proteins. When plants are subjected to adversity stress, a series of changes will occur in the hormone levels, thereby initiating or regulating certain physiological and biochemical processes related to stress resistance to complete the response to adversity. Moreover, some inorganic and organic osmotic substances such as Na^+^, K^+^, Cl^−^, proline (Pro), and soluble sugars may accumulate when plants encounter stresses. Further, under normal circumstances, the reactive oxygen species (ROS) are tightly controlled in plants, because plants have a reactive oxygen scavenging system, which keeps the production and removal of reactive oxygen species in a dynamic balance. This ROS includes hydrogen peroxide (H_2_O_2_), superoxide anion (O_2_·^-^), singlet oxygen (·O_2_), and hydroxyl radical (·OH) [3]. Under the condition of adversity, this balance is broken, and a large amount of active oxygen is produced. Active oxygen attacks the membrane system, causing changes in membrane lipid components and conformation of various enzymes on the membrane, loss of membrane selective permeability, leakage of electrolytes and certain small molecular organic substances, and disorder of mitochondria and chloroplast functions [1,4]. The active oxygen scavenging system mainly includes two types of substances: one is an enzymatic protection system composed of superoxide dismutase (SOD), peroxidase (POD), and catalase (CAT), etc.; the other is non-enzymatic antioxidants including reduced glutathione (GSH), carotenoids (Car), vitamin E, and other antioxidants [5]. Last but not least, some proteins such as NAC, WRKY, basic region/leucine zipper motif (bZTP), and salt overly sensitive1 (SOS1) participate in plant response to abiotic stresses [2,6].

Hydrogen sulfide (H_2_S) is a colorless, combustible, and hydrosoluble gas with an obvious smell of rotten eggs, which has been widely considered as the third gasotransmitter molecule besides nitric oxide (NO) and carbon monoxide (CO) [7]. The emission of H_2_S was studied a long time ago. In 1978, Wilson et al. (1978) firstly observed the emission of H_2_S in the leaves of cucumber (*Cucumis sativus* L.), squash and pumpkin (*Cucurbita pepo* L.), cantaloupe (*Cucumis melo* L.), maize, soybean (*Glycine max* L. Merr), and cotton (*Gossypium hirsutum* L.) [8]. Current studies show that H_2_S can be biosynthesized through a variety of enzymes such as cysteine synthase (CS), β-cyanoalanine synthase (CAS), L-cysteine desulfhydrase (LCD), D-cysteine desulfhydrase (DCD), and sulfite reductase (SiR) in mitochondria, cytosol, and chloroplast [9,10]. In mitochondria, H_2_S can be produced by CAS in the course of cyanide detoxification. The generation of H_2_S mainly occurs by inducing the activities of LCD and DCD from cysteine (Cys) in the cytosol, which is also accompanied by the formation of pyruvate and ammonia. SiR is the reaction catalyst in the photosynthetic sulfate-assimilation pathway which induces the release of H_2_S in the chloroplast [11,12]. Thus, endogenous H_2_S can be produced under the catalysis of the corresponding enzymes [8,9,10,11]. The changes in endogenous H_2_S level can influence cellular metabolisms, enzyme activities, and gene expressions, and thus modulate plant growth and development [5,13]. Therefore, H_2_S is widely considered as a signaling molecule within organic cells.

In the last few decades, increasing evidence has shown that H_2_S plays a vital role in the treatment of diseases for animals and humans, including cancer [13], burns [14], neurodegenerative diseases [15], and inflammation [16]. In addition, it is involved in many processes of growth and development in plants. It can influence the seed germination, root organogenesis, photosynthesis, stomatal movement, leaf senescence, fruit ripening and nodulation, and nitrogen fixation [17]. H_2_S can also enhance the plant’s tolerance to diverse biotic and abiotic stresses, such as bacterial and fungal pathogens, salinity, drought, heat, hyperosmotic, oxidative and heavy metal stresses, etc. [5,17,18,19].

**Figure 1:**
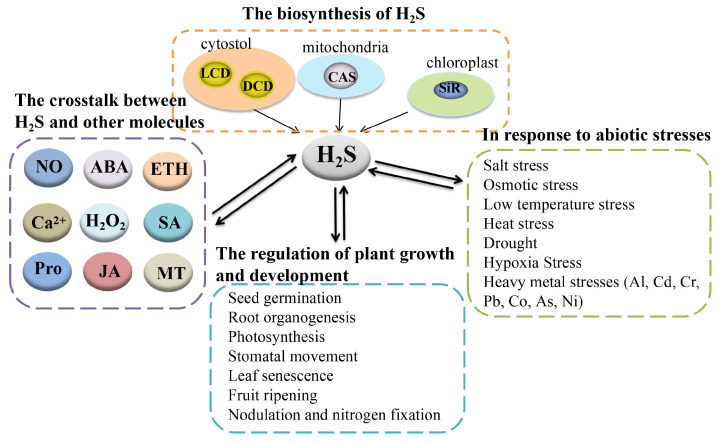
The summary of the biosynthesis of H_2_S, the crosstalk between H_2_S and other molecules, the regulation of plant growth and development, and the response to abiotic stresses by H_2_S. H_2_S, hydrogen sulfide; LCD, L-cysteine desulfhydrase; DCD, D-cysteine desulfhydrase; CAS, β-cyanoalanine synthase; SiR, sulfite reductase; NO, nitric oxide; ABA, abscisic acid; Ca^2+^, calcium ion; H_2_O_2_, hydrogen peroxide; SA, salicylic acid; JA, jasmonic acid; Pro, proline; MT, melatonin; Al, aluminum; Cd, cadmium; Cr, chromium; Pb, lead; Co, cobalt; As, arsenic; Ni, nickel.

As a gaseous signaling molecule, H_2_S can interact with other signal molecules to influence the growth and development of, and respond to abiotic stresses in, plants. Plenty of research demonstrates that H_2_S is involved in NO-alleviated salt stress and heavy metal stresses in the seedling roots of pea (*Pisum sativum* L. cv. Azad P-1) and barley (*Hordeum vulgare* L.), as well as the seeds of alfalfa (*Medicago sativa* L. cv. Victoria) [20,21,22]. Besides, some plant hormones such as abscisic acid (ABA), salicylic acid (SA), ethylene (ETH), jasmonic acid (JA), and melatonin (MT) could alleviate abiotic stresses together with H_2_S in the process of plant growth and development. Some ionic signals such as calcium ion (Ca^2+^) and H_2_S are interrelated under stresses [23]. Meanwhile, H_2_O_2_ and proline (Pro) have been reported to have a relationship with H_2_S under abiotic stresses during the process of plant growth [17,24,25]. Here, we comprehensively review the crosstalk between H_2_S and other signal molecules in response to abiotic stresses. Also, new research directions and future prospects in this area will be discussed in this review (Figure 1).

## 2. Crosstalk between H_2_S and NO in Response to Abiotic Stresses

NO is widely recognized as a gas transmitter in the regulation of seed germination, dormancy, stomatal aperture, adventitious root development, and photosynthesis in plants [26,27]. NO also takes part in many stress alleviation processes, such as heavy metal, extreme temperature, drought, salt, and UV-B radiation [4,28]. Moreover, the relationship between H_2_S and NO under different stress conditions has been explored at both the physiological and molecular levels, which remains a hot topic in plant science research in recently years. The obtained achievements in this field were collected and shown below.

### 2.1. Crosstalk between H_2_S and NO in Response to Heavy Metal Stress

There is considerable research on how H_2_S and NO interplay with each other in plants under heavy metal stress. In pea seedlings, As (V) reduced growth, photosynthesis capacity, and nitrogen content [29]. An application of exogenous NaHS alleviated As (V) toxicity by inducing H_2_S and NO generation. These results suggest a vital role of H_2_S in As (V) stress tolerance. Also, exogenous H_2_S and NO could reduce the influence of Cr (VI) toxicity in maize (*Zea mays* L.) in a similar manner [30]. Furthermore, H_2_S donor NaHS and NO donor sodium nitroprusside (SNP), rather than other derivatives, were found to specifically ameliorate Cd-induced oxidative damage in the root tissues of alfalfa seedlings [31]. This work further confirms that both H_2_S and NO may participate in alleviating heavy metal stress. In addition, the alleviation effects of NaHS and SNP were reversed by NO scavenger 2-(4-carboxyphenyl)-4,4,5,5-tetramethylimidazoline-1-oxyl-3-oxide potassium salt (cPTIO) [31], illustrating crosstalk between H_2_S and NO during the response to Cd stress. Another study in wheat (*Triticum aestivum* L.) obtained similar results that exogenous H_2_S might correlate with NO to enhance Co tolerance [32]. The above studies show that H_2_S may cooperate with the NO signal in managing different heavy metal stresses in plants.

**Figure 2:**
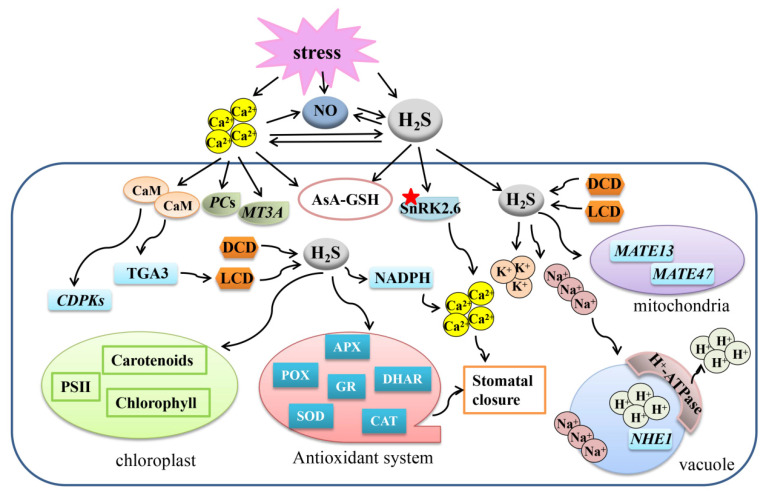
Overview for the mechanisms of the crosstalk between Ca^2+^, NO, and H_2_S to regulate plant response to abiotic stresses. A protein marked with a red asterisk means that the protein can be persulfided. Ca^2+^, calcium ion; NO, nitric oxide; H_2_S, hydrogen sulfide; LCD, L-cysteine desulfhydrase; DCD, D-cysteine desulfhydrase; APX, ascorbate peroxidase; SOD, superoxide dismutase; GR, glutathione reductase; POD, peroxidase; CAT, catalase; CaM, calmodulin; PCs, phytochelatin synthase; MT3A, metallothionein-like type 3; CDPKs, Ca^2+^-dependent protein kinases; AsA-GSH, ascorbate-glutathione cycle; DHAR, dehydroascorbate reductase; POD, peroxidase; CAT, catalase.

The pharmacological method of introducing specific scavengers into different experimental conditions was further employed to research the relationship between H_2_S and NO under heavy metal ion stress in plants. Cd stress was shown to induce a burst of endogenous NO and H_2_S in bermudagrass [*Cynodon dactylon* (L). Pers.] [33]. Moreover, exogenous NO donor SNP and H_2_S donor NaHS could improve Cd stress tolerance, while the positive roles of SNP and NaHS were specifically blocked by H_2_S scavenger hypotaurine (HT, C_2_H_7_NO_2_S), but not by NO scavenger cPTIO and H_2_S inhibitors potassium pyruvate (PP, C_3_H_3_KO_3_) and hydroxylamine (HA, H_3_NO). PP is regarded as the substrate of dehydrogenase. H_2_S could interact with the dehydrogenase. HA is an alkaline inorganic amine, which can react with the acid gas H_2_S; thereby, PP and HA are able to inhibit the production of endogenous H_2_S [33]. Thus, NO could activate the H_2_S signal in response to Cd stress, and maybe H_2_S is downstream of the NO signal. This phenomenon was further proved by the study of Al stress in soybean roots, in which NO modulated *GmMATE13* and *GmMATE47* gene expressions to enhance citrate secretion, and regulated PM H^+^-ATPase activity through regulating H_2_S biosynthesis and degradation [34]. H_2_S and NO improved Pb tolerance in *Sesamum indicum*, while the H_2_S-induced response was completely eliminated by NO scavenger cPTIO [35]. Meanwhile, only part of the effect conducted by NO was weakened by H_2_S scavenger HT. It seems that NO acts downstream of H_2_S or independent of H_2_S in conferring plant tolerance to Pd stress. More recently, the downstream role of NO in cooperation with H_2_S was also discovered in pepper (*Capsicum annuum* L.) and wheat under Cd stress [36,37]. From the numerous studies of H_2_S and NO, a hypothesis may be drawn that there exists a two-side signal cascades mechanism between H_2_S and NO in mediating heavy metal damage (Figure 2).

### 2.2. Crosstalk between H_2_S and NO in Response to Salt Stress

It has long been recognized that H_2_S and NO participate in alleviating salt stress in different plant species. Salt treatment (conducted by NaCl) could increase endogenous H_2_S and NO generation in the leaves of *Nicotiana tabacum* L. cv. Havana by increasing L-Cys and L-Arg contents and enhancing H_2_S and NO biosynthesis enzyme activities [38]. Then, H_2_S and NO help plants to cope with oxidative stress induced by salinity. These results suggest that both H_2_S and NO contribute to enhancing salt tolerance. Moreover, H_2_S donor NaHS and NO donor SNP relieved the inhibition of seed germination under salt stress in alfalfa through reestablishing ion homeostasis and maintaining activities of antioxidant enzymes [39]. The attenuation effect of salinity damage by H_2_S was reversed by NO scavenger cPTIO, suggesting that H_2_S enhanced salt tolerance through the NO pathway [39]. Another report discovered a similar relationship between H_2_S and NO in rescuing salt-induced inhibition of plant growth by regulating ion homeostasis [22].

The relationship between H_2_S and NO in salt resistance is still puzzled. It has been found that NO accumulation occurred ahead of H_2_S, however, H_2_S could not stimulate NO accumulation during the initial stage in salt-treated tomato (*Solanum lycopersicum*) roots [40]. The results above illustrate that H_2_S acts downstream of NO under salt stress, and may further induce NO production to strengthen the signal cascade in a feedback manner (Figure 2). In addition, H_2_S and NO may act downstream of MT to alleviate salt stress in pepper seedlings [41].

### 2.3. Crosstalk between H_2_S and NO in Response to Other Stresses

There also exists multiple pieces of evidence that H_2_S and NO cooperate with each other in heat, drought, osmotic, and flooding stresses. The pretreatment of exogenous NO enhanced the survival rate of maize seedlings under heat stress, and NO increased H_2_S content [42]. Furthermore, NO-induced heat tolerance was eliminated by H_2_S synthesis inhibitors and a H_2_S scavenger [42], indicating that H_2_S may act downstream of the NO signal in NO-induced heat tolerance. Later, another study discovered that SNP treatment facilitated the survival of submerged maize by enhancing the antioxidant system and regulating ROS content, elevating intracellular Ca^2+^ content and ADH activity, and increasing expressions of hypoxia-induced genes in maize seedling roots [43]. Moreover, SNP induced endogenous H_2_S generation, and H_2_S increased the NO-enhanced acquisition of tolerance to flooding-induced hypoxia in maize seedling roots [43], suggesting an analogical pattern of H_2_S and NO signal cascades in relieving heat and hypoxia stresses.

H_2_S may act as a downstream component of NO in ethylene-induced stomatal closure in *Vicia faba* L. [44]. Also, NO represented downstream of H_2_S in ABA-triggered stomatal closure, which may suggest a paradoxical relationship between H_2_S and NO under drought condition [45]. As for osmotic stress in wheat seedlings, the application of exogenous NO markedly improved H_2_S synthesis enzymes LCD and DCD, as well as enhancing the activity of *O*-acetylserine (thiol)lyase (OAS-TL) to modulate Cys homeostasis [46]. On the other hand, NO scavenger cPTIO and H_2_S scavenger HT invalidated the effect of NO on endogenous H_2_S levels and Cys homeostasis in wheat [46]. Thus, both H_2_S and NO could contribute to reinforcing osmotic tolerance and direct stomatal closure, though the concrete mechanism is largely unknown.

The H_2_S donor GYY4137 released a less severe H_2_S shock and a more prolonged H_2_S flux; however, it decreased NO accumulation in guard cells of *A*. *thaliana* leaves, in accordance with another type of H_2_S donor, NaHS [47]. In *Medicago sativa*, pretreatment with NOSH or NOSH-aspirin, the novel donors, which can donate NO and H_2_S simultaneously to plants, could enhance plant tolerance to drought stress and improve the recovery phenotype followed by rewatering [48]. Considering the cooperative relationship between H_2_S and NO, acting as signal molecules in retarding environmental damages, NOSH or NOSH-aspirin seems to be more favorable compared with NaHS and GYY4137 when used in plant guard cells, however, the effect and dosage have yet to be demonstrated (Figure 2).

## 3. Crosstalk between H_2_S and ABA in Response to Abiotic Stresses

ABA has long been recognized as a significant phytohormone with the function of regulating plant growth, development processes, and responses to diverse environmental stresses [49]. Within drought stress, ABA may take a central role in endogenous physiological processes, including stomatal movement [50,51]. Stomata are pores of plant aerial tissues and consist of a pair of guard cells. The stomatal aperture can be modulated by these specialized cells to respond to external and internal stimuli [52]. Within the past 10 years, the research of H_2_S and ABA crosstalk in augmenting plant tolerance to abiotic stresses has always come along with the regulation mechanism of stomatal movement.

### 3.1. Crosstalk between H_2_S and ABA in Response to Abiotic Stresses through Regulating Stomatal Closure

H_2_S cooperates with ABA in modulating the stomatal aperture, which has long been reported since [53] found that exogenous H_2_S regulated stomatal movement and enhanced leaf relative water content (RWC) to strengthen plant drought tolerance in *Arabidopsis thaliana*. Furthermore, scavenging H_2_S by HT or inhibiting H_2_S biosynthesis partially blocked ABA-dependent stomatal closure through regulating ATP-binding cassette transporters [53]. Similarly, pretreatment with H_2_S could considerably enhance rice’s tolerance to drought stress by decreasing lipid peroxidation, maintaining antioxidant system activation, and improving ABA biosynthesis [54]. The results above affirm a role of H_2_S in ABA signaling under environmental stresses. Furthermore, the stomatal aperture was enlarged in *lcd* mutant plants, causing a sensitive drought phenotype [55]. In addition, *LCD* expression and H_2_S generation were down-regulated in ABA-related mutants *aba3* and *abi1*, and NaHS application increased stomatal closure in these mutants [55]. Thus, H_2_S may regulate stomatal aperture in an ABA-dependent manner, and ABA may induce H_2_S biosynthesis under drought stress. Simultaneously, another report revealed that pretreatment of exogenous H_2_S enhanced wheat seedling tolerance to drought conditions through reinforcing antioxidant capacity [56]. Besides, the application of H_2_S modulated ABA metabolic pathway genes and up-regulated ABA receptors, indicating again that H_2_S alleviates drought stress, at least in part, through the ABA signaling pathway. Furthermore, exogenous ABA induced the endogenous H_2_S content under drought stress [56], illustrating a complex relationship between H_2_S and ABA signals in modulating drought stress.

Mitogen-activated protein kinases (MAPKs) belong to a crucial signaling molecule family which adjusts plants to multiple environmental stimuli [49]. In *A. thaliana*, drought stress fortified H_2_S generation and gene expression of MAPK, however, the induced MAPK expression was abolished in H_2_S synthesis double mutants *lcd des1* [57]. Further, the contributions of ABA to stomatal movements were also inhibited in *lcd des1* and *mpk4* mutants. In addition, H_2_S-enhanced stomatal closure was impaired in *slac1-3* mutants [57], in which SLAC1 is an S-type anion channel that responds to ABA signaling in stomatal closure [58]. A previous report announced that H_2_S could activate S-type anion currents via SLAC1 to induce stomatal closure [59]. In all, it could be proposed that H_2_S is involved in ABA-stimulated stomatal closure. Thus, MPK4 may act downstream of H_2_S, and H_2_S-MPK4 signal cascade is involved in ABA-stimulated stomatal closure in alleviating drought stress [57].

**Figure 3:**
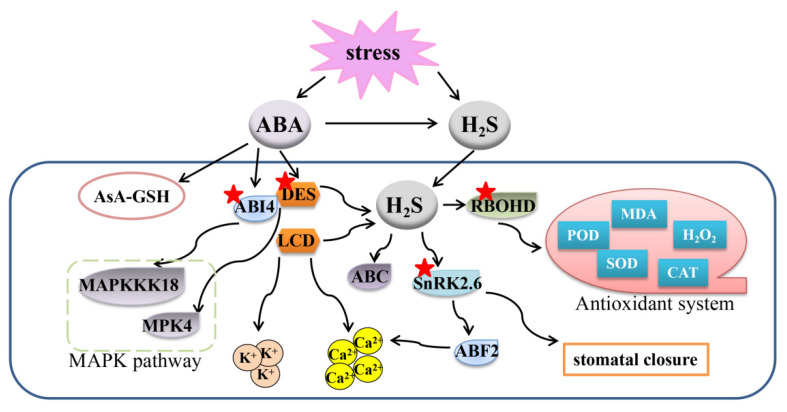
Overview of the mechanisms of the crosstalk between ABA and H_2_S to regulate plant response to abiotic stresses. A gene or protein marked with a red asterisk means that the protein can be persulfided. H_2_S, hydrogen sulfide; ABA, abscisic acid; ABF2, ABA response element-binding factor 2; AsA-GSH, ascorbate-glutathione cycle; SnRK2.6, snf1-related protein kinase 2.6; RBOHD, respiratory burst oxidase homolog protein d; MDA, malondialdehyde, ABI4, abscisic acid insensitive 4; MAPK, mitogen-activated protein kinase.

Osmotic stress adversely causes internal environmental disorder on account of the overproduction of ROS, which leads to a decrease in plant growth and productivity. Usually, plants resist osmotic stress by enhancing the antioxidant system and stimulating signal transductions [60]. Wheat could adjust itself to resisting osmotic stress by enhancing antioxidant systems and inducing H_2_S biosynthesis [61]. Furthermore, exogenous ABA induced AsA-GSH cycle activity, but H_2_S scavenger HT and synthesis inhibitor aminooxy acetic acid (AOA) reversed the activities mentioned above [61]. These results suggest that H_2_S induced by exogenous ABA is a signal that triggers the up-regulation of the AsA-GSH cycle under osmotic stress. Obviously, H_2_S takes part in ABA-related stomatal closure in response to different environmental stresses; however, the relationship between them is complicated (Figure 3).

### 3.2. Crosstalk between DES1/H_2_S and ABA in Response to Drought Stress through Regulating Protein Persulfidation

ABA could stimulate H_2_S generation under stresses, but how H_2_S synthesis enzyme DES1 contributes to the crosstalk between ABA and H_2_S is puzzled. Recently, by creating transgenic lines that expressed *DES1* in a tissue-specific pattern, it was found that the guard cell-specific DES1 was involved in ABA-induced physiological molecular responses [62]. ABA-induced DES1 expression and H_2_S production in guard cells were inhibited by H_2_S scavenger and restored by H_2_S donor [62]. The above genetic and pharmacological evidence further confirmed the hypothesis that DES1 is a unique component in ABA signaling in guard cells, and guard cell in situ DES1, together with H_2_S, participates in ABA-guided stomatal closure [63].

Excitingly, another report discovered that the ABA signal was, in turn, commanded by H_2_S-induced persulfidation of Open stomata 1 (OST1)/Snf1-related protein kinase 2.6 (SnRK2.6) on Cys131 and Cys137 residues in *A. thaliana* [64]. The persulfidated SnRK2.6 then interacted with ABA response element-binding factor 2 (ABF2), an ABA downstream protein, to modulate stomatal movement. Also, ABA was detected to induce DES1 and DCD expressions within 5–30 min previously [63,65], which suggests that the accumulation of H_2_S by ABA is ahead of the occurrence of protein persulfidation. Together with the works above, a hypothesis that ABA induces H_2_S accumulation, which further persulfidates SnRK2.6 continuously to promote ABA signaling in guard cells, would be proposed. The persulfidated SnRK2.6 then enhanced ABA- and H_2_S-induced Ca^2+^ influx, which subsequently caused stomatal closure through the inhibition of inward K^+^ channels and activation of outward anion channels [66]. To be encouraged continually, the DES1/H_2_S-triggered persulfidation mechanism in ABA-regulated stomatal movement has been confirmed in another two reports [67,68]. One of their works found that ABA triggered DES1 accumulation, and DES1 auto-presulfidated at Cys44 and Cys205 in a redox-dependent fashion, causing a trigger of transient H_2_S overproduction in guard cells [67]. They also found that the sustained DES1/H_2_S drove persulfidation of the NADPH oxidase respiratory burst oxidase homolog protein d (RBOHD) at Cys825 and Cys890 to strengthen its ability to introduce a ROS burst, which in turn induced stomatal closure [67]. Together, this work suggests that H_2_S-guided persulfidantion of DES1 and RBOHD may form a negative feedback loop that fine-tunes guard cell redox homeostasis and ABA signaling. Abscisic acid insensitive 4 (ABI4) could also be persulfidated by DES1 at Cys250 in vitro and in vivo, and served as a downstream target of H_2_S in plant’s response to ABA under stress conditions [68]. In addition, DES1-linked persulfication of ABI4 induced *MPAKKK18* transactivation through binding to the CE1 motif in the *MAPKKK18* promoter, which further enlarged the MAPK signaling cascade induced by ABA. Meanwhile, ABI4 could bind to the *DES1* promoter and, in turn, activate its transcription, forming a DES1-ABI4 loop to fine-tune ABA-MAPK signals [68]. The results above illustrate a redox-based protein persulfidation mechanism within the crosstalk between H_2_S- and ABA-involved stomatal movement [69]. Further work may focus on the molecular mechanisms of persulfidation and other post-translational modification events in H_2_S-regulated ABA signaling in guard cells (Figure 3).

## 4. Crosstalk between H_2_S and Ca^2+^ in Response to Abiotic Stresses

Ca^2+^ is another well-known second messenger in plant cells with the function of regulating intracellular physiological and biochemical processes, including alleviating abiotic stresses. Calmodulin (CaM) is a receptor protein in calcium signal transduction, and its main function is to perceive the volatility of intracellular calcium ions [10,70]. Recent studies uncovered a new signal transduction pattern in which Ca^2+^ and H_2_S cooperate to help plants resist environmental stresses.

### 4.1. Crosstalk between H_2_S and Ca^2+^ in Response to Heavy Metal Stress

Ca^2+^ influx was found to participate in restraining heavy metal contamination together with H_2_S signal cascade. H_2_S synthesis inhibitor and Ca^2+^ chelators aggravated the toxic phenotypes of foxtail millet (*Setaria italica*) exposed to Cr (VI) damage, demonstrating the involvement of H_2_S and Ca^2+^ signals during this process [71]. Furthermore, Ca^2+^ enhanced the expressions of heavy metal chelator biosynthesis genes *Metallothionein-like type 3* (*MT3A*) and *Phytochelatin Synthase* (*PCS*) and activated the antioxidant system, which was partially dependent on the H_2_S signal [71], indicating a downstream role of H_2_S in Ca^2+^ signaling. A later report in *A. thaliana* further discovered that the expression of H_2_S synthesis enzyme LCD was increased through a Ca^2+^/calmodulin 2 (CaM2)-directed pathway, which may explain the generation of H_2_S in the defense of plants against the Cr (VI) toxic condition [72,73]. The detailed mechanism was that the extracellular Cr (VI) stimulated Ca^2+^ influx, and the CaM2 protein then bound Ca^2+^ and interacted with the bZIP transcription factor TGA3, which further reinforced *LCD* gene expression and enhanced H_2_S production [72]. Ca^2+^ and H_2_S donor NaHS induced AsA-GSH cycle, redox homeostasis, and *Ca^2+^-dependent protein kinase* (*CDPK*) and *Phytochelatins* (*PCs*) genes expressions under Ni toxicity in zucchini seedlings [74]. In addition, H_2_S scavenger HT inhibited H_2_S accumulation induced by Ca^2+^, and Ca^2+^ chelator ethylene glycol-bis(b-aminoethylether)-N,N,N’,N’-tetra-acetic acid (EGTA) eliminated the impacts of seed priming induced by NaHS [74]. Thus, Ca^2+^ and H_2_S may manifest a two-side crosstalk in inoculating plants against heavy metal conditions. The relationship between NO and H_2_S has been discussed in another part of the present article, and it was put forward that Ca^2+^, in association with NO and H_2_S, improved chlorophyll metabolism, photosynthesis, carbohydrate accumulation, and maintained redox homeostasis in *Vigna radiata* under Cd stress [32]. The study also discovered that NO scavenger cPTIO could reduce Ca^2+^ content, and that EGTA reduced H_2_S content and altered Ca^2+^-dependent LCD and DCD enzyme activities, but that HT could not considerably reduce Ca^2+^ content [32]. Therefore, Ca^2+^, as a downstream signal of NO, may act in a two-side crosstalk pattern with H_2_S during plants’ adjustment to heavy mental contamination (Figure 2).

### 4.2. Crosstalk between H_2_S and Ca^2+^ in the Regulation of Stomatal Closure

Stomatal closure is an important physiological process under stress conditions; thus, the role of Ca^2+^ in stomatal closure was also summarized here. As mentioned above, H_2_S contributed to regulate S-type anion channel activation in guard cells, and this process was correlated with the SnRK2.6 function and the level of cytosolic free Ca^2+^ [59]. Further, H_2_S induced the Ca^2+^ influx in guard cells by stimulating the accumulation of ROS [75]. H_2_S triggered the persulfidation of SnRK2.6, and the persulfidated SnRK2.6 enhanced ABA- and H_2_S-induced Ca^2+^ influx, which subsequently caused stomatal closure [64]. Therefore, Ca^2+^ may function downstream of H_2_S-driven stomatal closure in a redox- and post-translational persulfidation-dependent manner (Figure 2).

### 4.3. Crosstalk between H_2_S and Ca^2+^ in Response to Other Stresses

As signal messengers, the crosstalk between H_2_S and Ca^2+^ has also been validated in many kinds of other stress conditions. Pretreating with H_2_S enhanced the heat tolerance of tobacco (*Nicotiana tabacum* L.) suspension-cultured cells by inhibiting electrolyte leakage and MDA accumulation, and exogenous Ca^2+^ and its ionophore A23187 intensified these effects [76]. However, H_2_S-induced heat tolerance was restrained by the application of Ca^2+^ chelator EGTA, as well as CaM antagonists chlorpromazine (CPZ) and trifluoperazine (TFP), illustrating a role of Ca^2+^ and CaM in H_2_S-triggered heat tolerance [76]. Afterward, another study announced that exogenous H_2_S enhanced the heat resistance of wheat coleoptiles through strengthening antioxidant enzyme activities in a Ca^2+^-dependent manner [77]. Thus, Ca^2+^ and CaM participate in H_2_S-induced heat tolerance in plants.

As for K^+^ deficiency under NaCl stress in *Vigna radiata* seedlings, Ca^2+^ increased endogenous H_2_S generation, and Ca^2+^ and H_2_S then cooperated with each other to induce an Na^+^/H^+^ antiport system and antioxidant defense [78]. Considering another result that adding of Ca^2+^-chelator EGTA and H_2_S scavenger HT reversed the effects of Ca^2+^ [78], a hypothesis may be drawn that H_2_S acts downstream during Ca^2+^-mediated plant adaptive responses to NaCl stress (Figure 2).

## 5. Crosstalk between H_2_S and H_2_O_2_ in Response to Abiotic Stresses

H_2_O_2_ is a colorless transparent liquid and crucial signaling molecule. Various studies have shown that H_2_O_2_ plays important roles in seed germination, stomatal movement, shoot and root development, pollination, and fruit ripening [79]. Also, it can modulate the plant growth and development under abiotic stresses [80]. The crosstalk between H_2_S and H_2_O_2_ under stress has been studied in recent years.

### 5.1. Crosstalk between H_2_S and H_2_O_2_ in Response to Heavy Metal Stress

**Figure 4:**
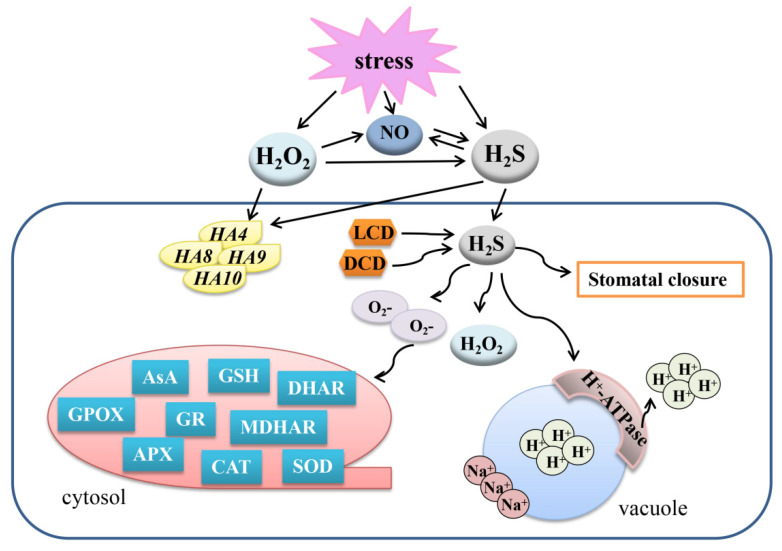
Overview of the mechanisms of the crosstalk between H_2_S, NO, and H_2_O_2_ to regulate plant response to abiotic stresses. H_2_S, hydrogen sulfide; H_2_O_2_, hydrogen peroxide; NO, nitric oxide; LCD, L-cysteine desulfhydrase; DCD, D-cysteine desulfhydrase; AsA, ascorbic acid; GSH, glutathione; GR, glutathione reductase; APX, ascorbate peroxidase; GPOX, guaiacol peroxidase; CAT, catalase; SOD, superoxide dismutase; DHAR, dehydroascorbate reductase; MDHAR, monodehydroascorbate reductase.

Cd stress could regulate the homeostasis of ROS and promote oxidative injury, which may cause cell death [81]. Cd could decrease vacuolar H^+^-ATPase activity, which was able to generate a proton gradient across the vacuolar membrane [82]. Under high Cd concentration stress, H_2_O_2_ and O_2_·^-^ significantly enhanced and triggered the oxidative injury, thus resulting in cell death in *Brassica rapa* root tips [81]. However, when *B. rapa* was exposed to low concentration Cd stress, the transcript levels of H_2_S biosynthesis-related genes *LCD* and *DCD* were significantly increased. Simultaneously, H_2_O_2_ had a remarkable increase and O_2_·^-^ went down, whereas H_2_S biosynthesis inhibitor or H_2_S scavenger reversed the positive effects, indicating a role of H_2_S in alleviating low Cd stress by adjusting the balance between H_2_O_2_ and O_2_·^−^ [81]. H_2_S donor NaHS treatment increased the photosynthetic fluorescence parameters in cotyledons of cucumber (*C. sativus* L. var. Wisconsin) seedling roots exposed to 100 μM CdCl_2_ for 24 h [82]. In addition, both the enhancement of H_2_O_2_ content and the decline in H_2_S content in roots decreased vacuolar H^+^-ATPase activity under Cd stress. Further, the increase in H_2_S content in root tissue by exogenous H_2_O_2_ had nothing to do with the desulfurization enzyme activity. Exogenous H_2_S remarkably enhanced the NADPH oxidase activity and the relative gene expression; however, it did not have an effect on the accumulation of H_2_O_2_ in cucumber roots under Cd stress [82]. Hence, H_2_S content might be partially enhanced through the H_2_O_2_/NADPH oxidase-induced pathway, independent of desulfhydrase activity (Figure 4).

### 5.2. Crosstalk between H_2_S and H_2_O_2_ in Response to Salt Stress

H_2_S donor NaHS could enhance the activity of PM H^+^-ATPase under salt or low-temperature stress in cucumber, and the transcript levels of the plasma membrane proton pump-related genes including *CsHA2*, *CsH4*, *CsH8*, *CsH9*, and *CsHA10* were also increased [83]. However, NO and H_2_O_2_ only enhanced the expression of *CsHA1*. Therefore, H_2_S, NO, and H_2_O_2_ could resist the salt stress by regulating the plasma membrane proton pump at different standards. Usually, salt stress could induce stomata closure. However, the H_2_S scavengers HT, AOA, hydroxylamine (NH_2_OH), potassium pyruvate (C_3_H_3_KO_3_), ammonia (NH_3_), H_2_O_2_, ascorbic acid (AsA), CAT, and diphenyl iodide (DPI) suppressed the closure of stomata in *V. faba* L. [44], suggesting that both H_2_S and H_2_O_2_ could regulate stomatal movement under salt stress. Furthermore, endogenous H_2_S and H_2_O_2_ accumulation and the activities of LCD and DCD were enhanced by salt treatment in guard cells. Nevertheless, these effects were inhibited by H_2_O_2_ and H_2_S scavengers. Exogenous H_2_O_2_ scavengers prevented the increase in endogenous H_2_S level as well as the stomatal closure; however, H_2_O_2_ generation was barely influenced with the application of H_2_S scavengers in guard cells responding to salt stress [44]. Hence, H_2_S may act as the downstream of H_2_O_2_-alleviated salt stress (Figure 4).

### 5.3. Crosstalk between H_2_S and H_2_O_2_ in Response to Drought Stress

Drought stress is one of the most serious abiotic stresses in the world. Treatment by spermidine (Spd) remarkably enhanced H_2_S production and activities of antioxidant enzymes [SOD, CAT, guaiacol peroxidase (GPOX), APX, GR, dehydroascorbate reductase (DHAR), and monodehydroascorbate reductase (MDHAR)] in white clover (*Trifolium repens*) under dehydration conditions [84]. Furthermore, NO and H_2_S scavengers could not reduce the generation of H_2_O_2_ induced by Spd, but H_2_O_2_ scavengers could effectively inhibit the increase of NO and H_2_S induced by Spd. The H_2_S signal induced by Spd was also significantly inhibited by NO scavenger [84]. Hence, in response to dehydration, H_2_S may be the downstream signaling molecule to interact with NO and H_2_O_2_ (Figure 4).

### 5.4. Crosstalk between H_2_S and H_2_O_2_ in Response to Other Stresses

UV-B is a common stress in practical agricultural production. When plants encounter the UV-B stress, the levels of electrolyte leakage, MDA, and ultraviolet absorbing compounds decreased, and the activities of antioxidant enzymes, GSH, and AsA also declined [85]. However, exogenous H_2_S, H_2_O_2_, and putrescence (Put) could alleviate the negative effects of UV-B stress. The protective role of Put in UV-B radiation damage was reduced by the inhibitors of H_2_S, H_2_O_2_, and Put [86]. Moreover, the level of H_2_O_2_ was increased by exogenous H_2_S, and the enhanced H_2_O_2_ promoted the accumulation of UV absorbing compounds in hulless barley (*H. vulgare* L. var. nude, Kunlun-12) seedlings, thus preserving the steady state of oxidation-reduction under UV-B stress and improving its UV-B tolerance [86].

In addition, extreme temperature is a key factor which influences plant growth and development. H_2_S, NO, and H_2_O_2_ had a significant impact in response to low temperature (10 °C) by modulating the plasma membrane proton pump in cucumber roots [83]. Moreover, H_2_O_2_ treatment could improve the heat resistance in maize (*Z. mays* L., Huidan No. 4) seedlings, and this effect could be strengthened by NO and H_2_S donors but abolished by NO and H_2_S scavengers or synthesis inhibitors [87]. It seems that NO and H_2_S act downstream of H_2_O_2_ in the acquisition of heat resistance in plants (Figure 4).

## 6. Crosstalk between H_2_S and Other Signal Molecules in Response to Abiotic Stresses

In recent years, many kinds of signal transmitters have emerged to regulate plant growth and development, and to acclimate to environment changes. The protective role of H_2_S related to these signal molecules such as SA, ETH, JA, Pro, and MT (mentioned in another part of the article) under toxic environment in plants has also been explored to some extent.

### 6.1. Crosstalk between H_2_S and SA in Response to Abiotic Stresses

**Figure 5:**
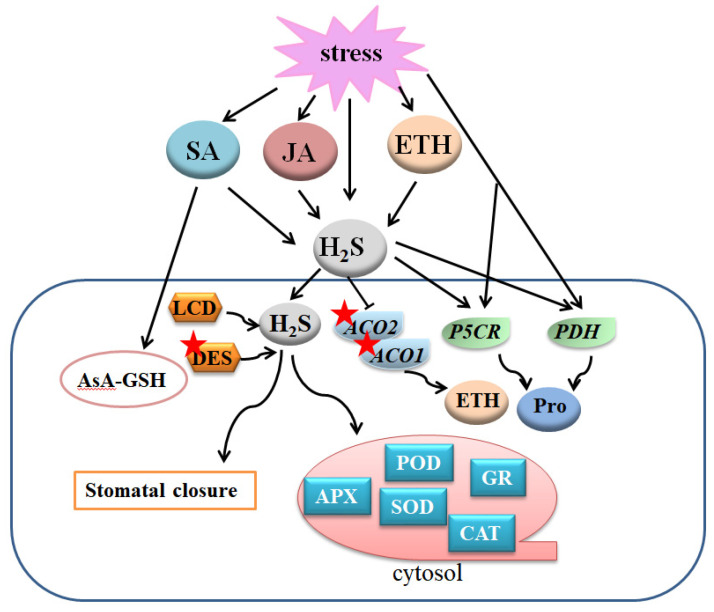
Overview of the mechanisms of the crosstalk between H_2_S and JA, SA, ETH, and Pro to regulate plant response to abiotic stresses. A gene or protein marked with a red asterisk means that the protein can be persulfided. H_2_S, hydrogen sulfide; LCD, L-cysteine desulfhydrase; DES, desulfhydrase; SA, salicylic acid; JA, jasmonic acid; ETH, ethylene; Pro, proline; APX, ascorbate peroxidase; SOD, superoxide dismutase; GR, glutathione reductase; POD, peroxidase; CAT, catalase; ACO1, 1-aminocyclopropane-1-carboxylic acid oxidase 1; ACO2, 1-aminocyclopropane-1-carboxylic acid oxidase 2; P5CR, proline-5-carboxylate reductase; PDH, proline dehydrogenase.

SA has long been recognized as a pivotal signal messenger, manifesting multiple functions in defending plant disease and adverse environmental conditions. Endogenous SA biosynthesis is mainly proceeded in the cytoplasm through the phenylalanine route by phenylalanine ammonia lyase (PAL) and benzoic-acid-2-hydroxylase (BA2H) [10,88,89]. SA and H_2_S enhanced heat tolerance by strengthening the activities of antioxidant enzymes and increasing osmolyte content in maize seedlings [90]. Further, SA induced endogenous H_2_S generation by enhancing the activity of H_2_S synthesis enzyme DES [91]. While the increase in SA production and the relative enzyme activities of PAL and BA2H were rarely influenced by H_2_S, this downstream role of H_2_S in SA-induced stress responses was also similarly reported in Cd tolerance in *A. thaliana* [92]. Thus, the positive role of SA under the stress condition is partially dependent on H_2_S. Pb stress accelerated endogenous H_2_S production [35]. Moreover, SA improved enzyme activities of the AsA-GSH cycle system in pepper under Pb stress [93]. In addition, exogenous SA enhanced the H_2_S content, which was further reinforced by H_2_S donor NaHS. It seems that SA triggers endogenous H_2_S accumulation, which further regulates the AsA-GSH cycle to resist Pb toxicity (Figure 5).

### 6.2. Crosstalk between H_2_S and ETH in Response to Abiotic Stresses

Ethylene induced H_2_S biosynthesis in guard cells in tomatoes under osmotic stress [94]. Moreover, the effect of ethylene on resisting osmotic stress was reversed by H_2_S scavenger HT or H_2_S synthetic inhibitor PAG, suggesting a downstream component of H_2_S in ethylene-triggered stomatal closure under osmotic stress. Further, H_2_S induced the persulfidation of 1-aminocyclopropane-1-carboxylic acid oxidase1 (ACO1) and ACO2, and restrained their expressions. As a result, H_2_S negatively regulated ethylene generation in response to osmotic stress [94]. These results are parallel with a recently published mechanism of waterlogging damage resistance in peach (*Prunus persica* L. Batsch) seedlings [95], in which H_2_S restrained over-synthesis of ethylene as well as inhibited oxidative damage under waterlogging stress (Figure 5).

### 6.3. Crosstalk between H_2_S and JA in Response to Abiotic Stresses

JA is another phytohormone kind signal transmitter with extensive modulation functions in plant root elongation [96], anthocyanin accumulation and trichome initiation [97], stamen development and flowing [98], leaf senescence [99], and stress resistance [100]. A recent study announced a critical role of JA in inhibiting stomatal development in *A. thaliana* [101]. Furthermore, JA positively modified LCD activity and H_2_S production. The JA-deficient mutants represented a high stomatal density phenotype, which could be reversed by exogenous H_2_S, whereas the H_2_S synthesis-deficient mutants *lcd* displayed similar stomatal development phenotype as the JA-deficient mutants, which could be rescued by H_2_S donor NaHS but not by JA [102]. Thus, H_2_S may act as a downstream member of JA in stomatal development (Figure 5).

### 6.4. Crosstalk between H_2_S and Pro in Response to Abiotic Stresses

Pro is a kind of organic osmolyte with a wide distribution in plant cells. Previous studies have demonstrated the increase of Pro after the application of signal transmitter agents in defense of abiotic stresses [89,103,104]. Pretreatment with exogenous H_2_S increased endogenous Pro content, and the activities and transcription levels of proline-5-carboxylate reductase (P5CR) and proline dehydrogenase (PDH) in foxtail millet, whereas H_2_S scavenger or inhibitor reduced the above effects [105]. Moreover, the combined application of H_2_S and Pro resulted in preferable growth status, stomatal movement, and oxidative remission under stress conditions. These results indicate a cooperation of Pro and H_2_S under adverse environments (Figure 5).

## 7. Conclusions and Outlook

The disadvantageous environment conditions cause oxidative damage, ionic imbalance, and osmotic stress to plants, resulting in a weakened growth and development status. H_2_S can reinforce plant tolerance to these stresses through constructing a luxuriant crosstalk with other signal molecules, such as NO, ABA, Ca^2+^, H_2_O_2_, SA, ETH, JA, Pro, and MT. The genes regulated by H_2_S and other molecules under abiotic stress conditions are displayed in Table 1. There exists a legible clue that environmental stresses and various signal transmitters stimulate endogenous H_2_S generation and improve the activities of H_2_S synthesis enzymes under the stress condition. Meanwhile, H_2_S represents a feedback manner to enhance the signal cascades in inducing the accumulation of some signal messengers, especially NO, ABA, and Ca^2+^. In addition, the existence of DES1-related auto-persulfidation and persulfidation may be the reason for the extensive inspiration of its enzyme activity in different stress conditions. In summary, H_2_S acts as a downstream signal member in cooperation with ABA, H_2_O_2_, SA, ETH, JA, and MT, but an upstream signal member of Pro under stress condition. Nevertheless, the crosstalk between H_2_S, NO, and Ca^2+^ represents a two-side signal cascades manner, whereas relationships between H_2_S and other signal molecules vary on account of the specific stress pattern.

Multiple types of research need to be done to explore the point-to-point mechanism within the crosstalk between H_2_S and one single signal transducer under abiotic stress conditions. Firstly, the feedback molecular mechanism of H_2_S and NO, and the interactions within protein persulfidation, *S*-sulfhydration, and *S*-nitrosylation, remain unclear. Next, more post-translational modification proteins need to be discovered and identified that are triggered by H_2_S in ABA- or NO-dependent signal pathways under stress condition. Finally, new signal messengers related to H_2_S activity are waiting to be discovered.

## Figures and Tables

**Table 1:** Genes regulated by H_2_S and other molecules under abiotic stress conditions.

Crosstalk between H_2_S and other Molecules	Stresses	Plant Species	Tissue	Regulated Genes	References
H_2_S and NO	salt stress	*Medicago sativa*	seeds	*APX-1*, *APX-2,* and *Cu/Zn-SOD*	[39]
		*Hordeum vulgare* L.	seedlings	*HvHA*, *HvVHA-β*, *HvSOS1*, *HvVNHX2*, *HvAKT1* and *HvHAK4*	[22]
		*Solanum lycopersicum*	seedlings	*SlL-DES*, *SlCAS* and *SlCS*	[40]
	drought	*M. sativa* L.	leaves	*GST17*, *Cu/ZnSOD*, *FeSOD*, *NR*, cAPX, PIP	[48]
	hypoxia stress	*Zea mays* L.	seedlings	*P4H*, *ADH*, *CRT1*, *GS*, *CYP51* and *ME*	[43]
	cadmium stress	*M. sativa* L.	seedlings	*Cu/Zn–SOD*, *APX* and *POD*	[31]
	cobalt stress	*Triticum aestivum* L.	seedlings	*RbcL*	[32]
	aluminum stress	*Glycine max* L.	seedlings	*MATE13*, *MATE47*, *MATE58*, *MATE74*, *MATE79*, *MATE84*, and *MATE87*	[34]
H_2_S and ABA	drought	*Oryza sativa* L.	seedlings	*NCED2*, *NCED3*, *NCED5*, *AREB1*, *AREB8*, *bZIP23* and *LEA3*	[54]
		*Arabidopsis*	seedlings	*TPC1*, *GORK*, *SKOR*, *KCO1, MYP5, ACA9*, *ACA11*, *CAX1*, *SLAC1*, *AKT1A*, *KT2*, *KC1* and *KAT1*	[55]
		*T. aestivum* L.	leaves and roots	*TaZEP*, *TaNCED*, *TaAAO* and *TaSDR*	[56]
		*Arabidopsis thaliana*	-	*MAPK*s	[57]
	chromium stress	*A. thaliana*	seedlings	*LCD*	[72]
	nickel stress	*Cucurbita pepo* L.	seedlings	*CDPK* and *PCS1*	[74]
H_2_S and Ca^2+^	chromium stress	*Setaria italica*	seedlings	*MT3A*, *PCS*, *CaM*, *CBL* and *CDPK*	[71]
H_2_S-H_2_O_2_	cadmium stress	*Brassica rapa.*	seedlings	*Br_UPB1A*, *Br_UPB1B*↑; *Bra035235*, *Bra033551*, *Bra006423*, ra023639	[89]
	cadmium stress	*Cucumis sativus* L.	roots	*CsVHA-A*, *CsVHA-B*, *CsVHA-a1*, *CsVHA-a2*, *CsVHA-a3*, *CsVHA-c1*, *CsVHA-c2* and *CsVHA-c3*	[82]
H2S, NO and H_2_O_2_	salt or low temperature	*C. sativus* L.	roots	*CsHA1*, *CsHA2*, *CsH4*, *CsH8*, *CsH9* and *CsHA10*	[83]
	dehydration	*Trifolium repens*	seedlings	*bZIP37*, *bZIP107*, *DREB2*, *DREB4* and *WRKY108715*	[84]
H_2_S and ETH	osmotic stress	*S. lycopersicum*	seedlings	*LeACO1 and LeACO2*	[94]
H_2_S and Pro	cadmium stress	Foxtail millet	seedlings	*PDH* and *P5CR*	[105]

APX, ascorbate peroxidase; SOD, superoxide dismutase; HA, H^+^-ATPase; VNHX2, vacuolar Na^+^/H^+^ antiporter; VHA-β, H^+^-ATPase subunit β; HAK4, high-affinity K^+^ uptake system; L-DES, L-cysteine desulfhydrase; CAS, β-cyanoalanine synthase; CS, L-cysteine synthase; P4H, prolyl 4-hydroxylase; ADH, alcohol dehydrogenase; CRT1, calcium binding protein; CYP51, cytochrome P450 14a-sterol demethylase; GS, glutamate synthase 1; ME, NADP-dependent malic enzyme; POD, peroxidase; rbcL, rubisco large subunit; NCED, 9′-cis-epoxycarotenoid dioxygenase; TPC1, two pore segment channel 1; GORK, guard cell outward-rectifying Kþ channel; SKOR, SKI family transcriptional corepressor; KCO, outward-rectifying K^+^ channel; ACA, adenylyl cyclase-associated protein; CAX, calcium exchanger; SLAC1, slow anion channel associated 1; AKT, *Arabidopsis* potassium transporter; KC1, potassium channel 1; KAT1, potassium channel in *Arabidopsis thaliana* 1; ZEP, zeaxanthin epoxidase; AAO, abscisic aldehyde oxidase; SDR, short-chain dehydrogenase; MAPK, mitogen-activated protein kinase; LCD, L-cysteine desulfhydrase; CDPK, Ca^2+^-dependent protein kinase; PCS, phytochelatin; CaM, calmodulin; CBL, calcineurin B-like; ACO, 1-aminocyclopropane-1-carboxylic oxidase; PDH, proline dehydrogenase; P5CR, proline-5-carboxylate reductase.
